# Cell‐free DNA copy number variations in plasma from colorectal cancer patients

**DOI:** 10.1002/1878-0261.12077

**Published:** 2017-06-06

**Authors:** Jian Li, Rachel L. Dittmar, Shu Xia, Huijuan Zhang, Meijun Du, Chiang‐Ching Huang, Brooke R. Druliner, Lisa Boardman, Liang Wang

**Affiliations:** ^1^ Department of Gastroenterology Henan Provincial People's Hospital Zhengzhou University Zhengzhou China; ^2^ Department of Pathology and MCW Cancer Center Medical College of Wisconsin Milwaukee WI USA; ^3^ Department of Oncology Tongji Hospital of Tongji Medical College Huazhong University of Science and Technology Wuhan China; ^4^ Physical Examination Center Zhengzhou Seventh People's Hospital Zhengzhou China; ^5^ Department of Biostatistics University of Wisconsin Milwaukee WI USA; ^6^ Department of Oncology Mayo Clinic Rochester MN USA

**Keywords:** cell‐free DNA, colon cancer, copy number variation, next‐generation sequencing, survival

## Abstract

To evaluate the clinical utility of cell‐free DNA (cfDNA), we performed whole‐genome sequencing to systematically examine plasma cfDNA copy number variations (CNVs) in a cohort of patients with colorectal cancer (CRC,* n* = 80), polyps (*n* = 20), and healthy controls (*n* = 35). We initially compared cfDNA yield in 20 paired serum–plasma samples and observed significantly higher cfDNA concentration in serum (median = 81.20 ng, range 7.18–500 ng·mL^−1^) than in plasma (median = 5.09 ng, range 3.76–62.8 ng·mL^−1^) (*P* < 0.0001). However, tumor‐derived cfDNA content was significantly lower in serum than in matched plasma samples tested. With ~10 million reads per sample, the sequencing‐based copy number analysis showed common CNVs in multiple chromosomal regions, including amplifications on 1q, 8q, and 5q and deletions on 1p, 4q, 8p, 17p, 18q, and 22q. Copy number changes were also evident in genes critical to the cell cycle, DNA repair, and WNT signaling pathways. To evaluate whether cumulative copy number changes were associated with tumor stages, we calculated plasma genomic abnormality in colon cancer (PGA‐C) score by summing the most significant CNVs. The PGA‐C score showed predictive performance with an area under the curve from 0.54 to 0.84 for CRC stages I‐IV. Locus‐specific copy number analysis identified nine genomic regions where CNVs were significantly associated with survival in stage III‐IV CRC patients. A multivariate model using six of nine genomic regions demonstrated a significant association of high‐risk score with shorter survival (HR = 5.33, 95% CI = 6.76–94.44, *P* < 0.0001). Our study demonstrates the importance of using plasma (rather than serum) to test tumor‐related genomic variations. Plasma cfDNA‐based tests can capture tumor‐specific genetic changes and may provide a measurable classifier for assessing clinical outcomes in advanced CRC patients.

**Abbreviations**
AUC: area under the curve
cfDNA: cell‐free DNA
CNAM: copy number analysis method
CNV: copy number variation
CRC: colorectal cancer
FDR: false discovery rate
PGA‐C: plasma genomic abnormality in colorectal cancer
PGA: plasma genomic abnormality
ROC: receiver‐operating characteristic curve

## 1 Introduction

With a global incidence of 1.3 million cases and a disease‐specific mortality of about 33%, colorectal cancer (CRC) is a major health burden (Ferlay *et al*., 2015). Unfortunately, approximately 50% of CRC patients have occult or detectable distant metastases at the time of diagnosis, which minimizes the chance of cure by surgical intervention. To facilitate early detection and reduce metastasis‐related death, various approaches have been used, including CEA and CA19‐9 measurement (Forones and Tanaka, 1999), fecal occult blood testing, and computed tomography imaging. Due to the lack of sensitivity and specificity, application of these detection methods has been limited (Bretthauer, 2011; Chao and Gibbs, 2009; Vukobrat‐Bijedic *et al*., 2013). Additionally, colonoscopy‐based biopsies are invaluable tools for CRC diagnoses and prognoses. However, repeated biopsies are not recommended during treatment and surveillance for CRC (Issa, 2008). To address these limitations, circulating cell‐free DNA (cfDNA) in blood has been recently evaluated because it is a less‐invasive testing strategy for CRC patients. cfDNA analysis may provide direct evidence of residual disease, thus defining the group of patients at high risk for recurrence following surgery and other treatments (Tie *et al*., 2016). Similarly, the use of cfDNA has been reported to be superior to clinicopathological measures to guide adjuvant chemotherapy decisions for stage II CRC patients (Tie *et al*., 2016). This liquid biopsy approach for assessing the genetic makeup of solid tumors from a biofluid sample has been advocated for clinical care and for future oncological research (Heitzer *et al*., 2016).

Recent studies utilizing next‐generation sequencing of peripheral blood cfDNA confirm that genetic and genomic variations comprise a major mechanism driving carcinogenesis and drug resistance in CRC (Muzny *et al*., 2012; Wang *et al*., 2016). These genetic/genomic variations include cancer‐specific gene mutations as well as gross chromosome aberrations. Copy number variations (CNVs) are somatic changes that cause the gain or loss of DNA segments from a normal genome. Studies have shown that CNVs at different genomic locations are important in chromosomal instability‐related adenoma to carcinoma progression (Carvalho *et al*., 2009). More CNV gains have been reported in metastatic CRC than in nonmetastatic CRC (Diep *et al*., 2006). Thus, the CNVs play a critical role in CRC initiation and progression and may be involved in multiple signaling systems, such as RTK, PI3K, RAS, and WNT (Muzny *et al*., 2012; Wang *et al*., 2016). In this study, we performed whole‐genome sequencing‐based CNV analysis using plasma cfDNA derived from a well‐characterized clinical cohort including healthy controls, patients with colorectal polyps, and patients with stage I‐IV CRC.

## 2 Materials and methods

### 2.1 Patients and controls

Blood samples were obtained from two groups of patients, one with colorectal polyps and another with CRC. These patients were enrolled in a prospective study on the genetic role of CRC. Institutional review boards at both the Medical College of Wisconsin and Mayo Clinic approved this study. Blood samples from the healthy controls were also collected using the same protocol as patients. Control samples were age‐ and gender‐matched with the patient samples. All healthy controls were confirmed by colonoscopic examination. The controls and patients with polyps were healthy individuals without any history of cancer. They were recruited in their routine physical examination. For all patients with colon cancer, blood was collected before any type of treatment, usually 0–7 days before treatment procedures. For normal controls and polyps patients, blood was collected prior to colonoscopy. All participants provided written informed consent. Patients with CRC were assessed pathologically after surgery using the TNM system. All the clinicopathological data were retrieved from Mayo Clinic clinical database. The same patient blood samples were separated into the serum and plasma shortly after blood draw to compare the cfDNA yield and quality in serum and plasma. All plasma and serum samples were stored at −80 °C prior to DNA extraction.

### 2.2 cfDNA extraction and quantification

Cell‐free DNA extraction was published previously (Xia *et al*., 2015a,b). In brief, prior to DNA extraction, samples were removed from the freezer and thawed on ice. Samples were centrifuged at 3000***g*** at 4 °C for 10 min and then allowed to equilibrate to room temperature. cfDNA was extracted from 400 to 800 µL of plasma or serum using the DNA Blood Mini Kit (Qiagen, Valencia, CA, USA). Due to the excess volume, 2–4 aliquots of protease‐treated samples were run through the spin column separately. The protease incubation was increased from 10 min to 1 h to ensure complete removal of proteins. In the last step, 50 μL of all‐free water was applied to the column, incubated at room temperature for 3 min, and centrifuged. The eluents were then reapplied to the column, incubated for 3 min at room temperature, and centrifuged. Samples were quantified using a Qubit 2.0 Fluorometer (Life Technologies, Carlsbad, CA, USA).

### 2.3 Sequencing library preparation

DNA sequencing libraries were prepared using a ThruPLEX DNA‐seq Library Kit (Rubicon Genomics, Ann Arbor, MI, USA) per the manufacturer's instructions. 2 ng cfDNA was used for library preparation, which included end‐repair, addition of adapters, and 10 cycles of amplification. Following amplification, libraries were purified using a 1 : 1 ratio of sample to Agencourt AMPure XP Beads (Beckman Coulter, Indianapolis, IN, USA), in accordance with the instructions from Rubicon Genomics. Library DNA were eluted from the beads using 40 μL of IDTE pH 8.0 (IDT, Coralville, IA, USA) and quantified using a Qubit 2.0 Fluorometer. Sequencing library quality was assessed by a Bioanalyzer High Sensitivity DNA Analysis Kit and Chip (Agilent Technologies, Santa Clara, CA, USA). Library DNA were diluted to a concentration of 2 nm and then pooled for sequencing. An Illumina HiSeq 2500 (Illumina, Inc., San Diego, CA, USA) was used for single‐end 50‐basepair read.

### 2.4 Copy number variation calculation

Raw sequencing data (fastq files) were first mapped to the human genome (hg19) using SeqMan NGen12 (DNASTAR, Madison, WI, USA) and assembled in Partek Genomics Suite (St. Louis, MO, USA). The mapped reads were then binned into either 1 Mb (for overall copy number analysis) or 60 Kb (for locus‐specific copy number analysis) genomic windows (bins). After excluding sex chromosomes, all remaining reads were rescaled to 10 million reads. Read count in each genomic window was normalized to mean read count from 32 healthy controls. The resulting ratios were then transformed with log2 and adjusted for GC content (Diskin *et al*., 2008). The fully normalized log2 ratios in genomic windows were subjected to segmentation using the copy number analysis method (CNAM) algorithm (Golden Helix, Bozeman, MT, USA).

### 2.5 Plasma genome abnormality in colorectal cancer score

Plasma genomic abnormality (PGA) score was developed to measure tumor DNA burden in cfDNA (Xia *et al*., 2015a,b). In this study, mean values of genomic segments generated from the CNAM algorithm were used for plasma genome abnormality in colorectal cancer (PGA‐C) score calculation. Segment sizes were first evaluated to test PGA‐C score stability. The PGA‐C score was then calculated by summing the five most significant segment values (copy number changes) including both amplifications and deletions: PGA‐C = 100 × sum of absolute mean values from top five segments. A higher PGA‐C score indicates greater tumor‐specific DNA content in the cfDNA and thus higher tumor burden.

### 2.6 Statistical analysis

To compare copy number differences between study groups, genomic segments were summarized by their mean and standard deviation within the two study groups and analyses of covariance approaches were used. To evaluate whether PGA‐C score could differentiate between cases and controls, area under the receiver‐operating characteristic curve (AUC) analysis was used. This analysis examines all possible case–control pairs and measures the proportion of the time the statistical model predicts higher risk for the case (Zweig and Campbell, 1993). For stage III–IV patients, Cox proportional hazards regression and Kaplan–Meier survival curves were used to estimate association of copy number changes with overall survival. For the multivariate prediction model, the risk score was calculated by a linear combination of log2‐based segment values only, weighted by their estimated regression coefficients. To correct for multiple testing, *q*‐values to represent the false discovery rate (FDR) were used (Storey and Tibshirani, 2003). The segments with a FDR value ≤0.05 level were considered significant. All analyses were conducted using GraphPad (La Jolla, CA, USA) or Partek Genomics Suite (St. Louis, MO, USA).

## 3 Results

### 3.1 Patients’ clinical characteristics

We examined cfDNA for CNV analysis from a total of 135 subjects, namely 35 healthy controls, 20 patients with adenomatous polyps, and 80 patients with CRC. After quality checking for sequencing libraries and sequence read counts, we excluded three healthy controls and one stage II CRC patient. The remaining 131 subjects were used for all data analysis. Median follow‐up was 64.00 months (6.77–72.83 months) for all polyp and CRC patients. For the 20 polyp patients, seven patients had tubular adenoma (TA) with low‐grade dysplasia (LGD) and one had TA with high‐grade dysplasia (HGD). Five patients had tubulovillous adenoma (TVA) with LGD; three had TV with LGD and focal HGD; and one patient had both a TA and TVA with LGD. One patient had a sessile serrated polyp (SSP) with no dysplasia. Two patients had TA with LGD and a SSP; one patient had TA, TVA with LGD, and a SSP. The median age at diagnosis for polyp patients was 68 years and 52% were male. For 79 patients with CRC, the median age at diagnosis was 60.5 years and 49% were male. The median age at death was 69 years, and 47% of those who died were male. Twenty‐one of the cases arose in the right colon; 29 in the left colon; and 29 in the rectum. Seven stage II, 16 stage III, and 16 stage IV patients received postoperative chemotherapy with a 5 FU and oxaliplatin (FOLFOX)‐based regimen and six of these patients were also treated with postoperative radiation therapy. One stage I patient took oral 5 FU, and one stage IV patient was treated with PTK787, RAD001. Three stage III and one stage IV rectal cancer patients were also treated with neoadjuvant chemoradiotherapy. No patients received anti‐EGFR treatment. Clinical characteristics for controls, colorectal polyps, and CRC patients are presented in Table 1.

**Table 1:** Clinical characteristics of controls, colorectal polyps, and CRC patients

Characteristics	Controls (*n* = 32)	Polyps (*n* = 20)	CRC (*n* = 79)	Stage group			
				I (*n *= 20)	II (*n* = 19)	III (*n* = 20)	IV (*n* = 20)
Age (years)							
Median (Range)	52 (25–76)	67.5 (38–86)	60.5 (19–88)	58 (19–88)	70 (47–87)	70 (45–84)	57 (29–82)
Sex, *n* (%)							
Male (%)	16 (50)	10 (50)	39 (49)	10	10	9	10
Female (%)	16 (50)	10 (50)	40 (51)	10	9	11	10
BMI							
Median (Range)	27.1 (20.24–40.66)	26.74 (19.97–36.14)	26.54 (16.02–52.6)	26.41	27.09	27.63	26.11
Histology							
Adeno			74	20	19	16	19
Mucinous adeno			5	0	0	4	1
Histological grade							
Moderately differentiated			15	5	5	2	3
Poorly differentiated			60	14	12	17	17
Undifferentiated			2	0	2	0	0
Unknown			2	1	0	1	0
T stages							
T1			7	6	0	1	0
T2			20	14	0	6	0
T3			43	0	18	12	13
T4			8	0	1	1	6
Unknown			1	0	0	0	1
Lymph node metastasis							
Yes			37	0	0	20	17
No			40	20	19	0	1
Unknown			2	0	0	0	2
Distant metastasis							
M1			20	0	0	0	20
M0			59	20	19	20	0
Tumor location							
Right colon			21	5	6	5	5
Left colon			29	7	7	7	8
Rectum			29	8	6	8	7
Postop chemo/Radiation^a^							
Yes			40	1	7	16	16
No			37	19	12	4	2
Unknown			2	0	0	0	2

a Three stage III and one stage IV rectal cancer patients were also treated with neoadjuvant chemoradiotherapy.

### 3.2 cfDNA concentrations in plasma and matched serum

To compare the difference in cfDNA yield between serum and plasma, we extracted cfDNA from 20 serum–plasma pairs under the same DNA extraction conditions. Because input volumes were different, we normalized cfDNA yields to ng per mL of plasma or serum. Fluorometer‐based DNA quantification showed significant cfDNA yield difference between plasma and matched serum samples. Normalized cfDNA yield was an average of 109.29 ng (median = 81.20 ng, range 7.18–500 ng) per mL serum and 10.19 ng (median = 5.09 ng, range 3.76–62.8 ng) per mL plasma (Fig. S1A). Clearly, the cfDNA concentration was over 10‐fold lower in plasma than in matched serum samples (*P* = 0.0011). We also compared plasma cfDNA concentration among different disease stages and found significantly increased concentration in patients with stage IV CRC when compared to healthy controls (*P* = 0.0082). However, overall cfDNA concentrations in plasma were relatively stable in patients with CRC stages I‐III, patients with polyps, and healthy controls (Fig. S1B).

### 3.3 Sequencing library quality

To ensure high quality of these sequencing data, we performed quality controls during sequencing library preparation and in all sequencing data. For all sequencing libraries, we measured library DNA sizes using Agilent Bioanalyzer (Santa Clara, CA, USA). After adding adaptor sequences, the sequencing library showed multiple peak bands with the smallest peak size at 300–310 bp because cfDNA is dominantly derived from apoptotic cells where the nuclear DNA is degraded into smaller fragments reflecting the size of nucleosomes (Fig. S2). We also evaluated sequencing quality by assessing total sequence reads and mappable reads. From this analysis, we excluded four samples due to low read counts (<3 millions of raw reads). Among the remaining 131 samples, we received approximately 10.30 million raw reads (range from 3.30 to 32.83) and 9.56 million mappable reads (range from 3.08 to 30.10) on average for each library. Correspondingly, the average coverage was 0.16× (range from 0.05 to 0.50×) and mappable reads accounted for an average of 92.75% (range from 89.68 to 94.12%) of raw reads. Detailed statistics is listed in Table S1. To test the reproducibility of the copy number analysis method, we prepared technical replicates (duplicated libraries) in six samples (one from each group including control, polyps, stages 1–IV). This analysis showed that duplicate samples were clustered together (Fig. S3), demonstrating consistency of the copy number analysis method using low input of cfDNA.

### 3.4 Tumor‐specific cfDNA content in plasma and matched serum

**Figure 1:**
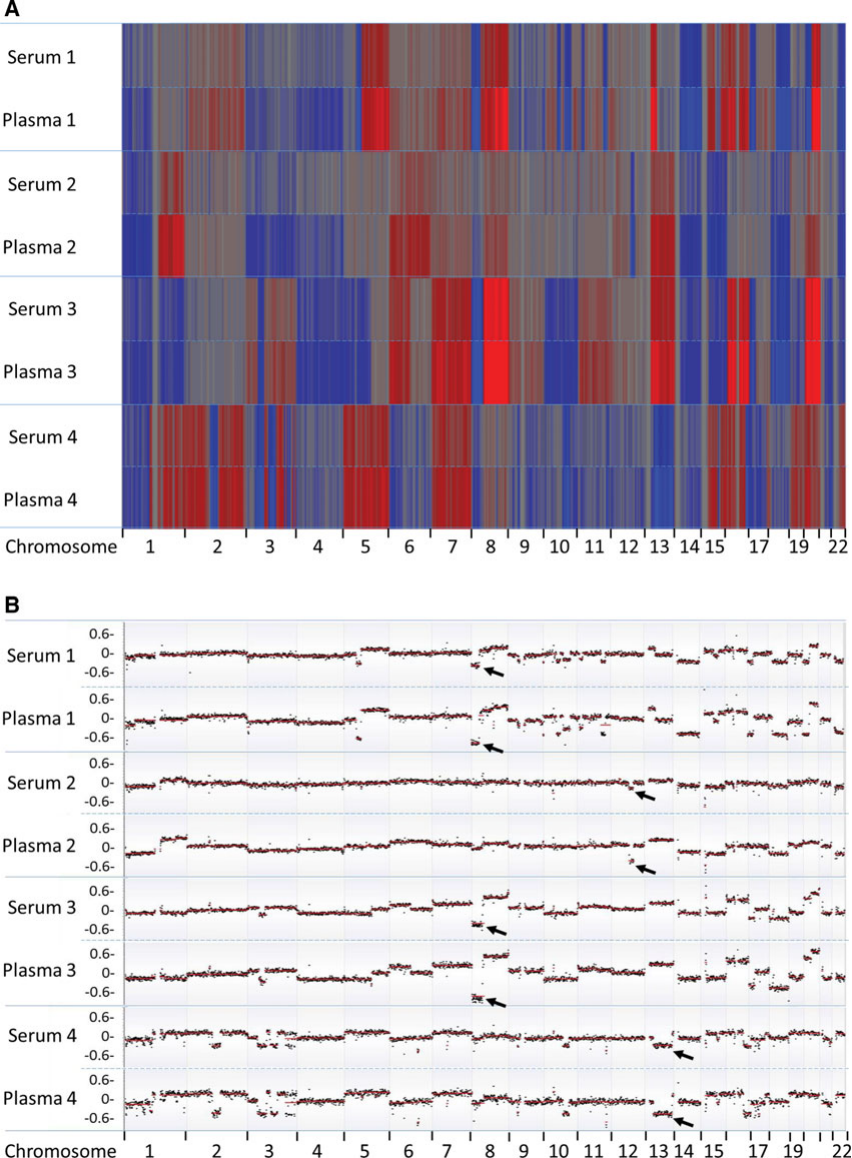
Comparison of copy number changes between four pairs of serum and plasma. (A) Heatmap of log2 ratio in 1‐Mb genomic window shows higher tumor‐specific cfDNA in plasma than in serum. Red color represents copy number gain, while blue represents loss. Intensity of the color is proportional to the value of log2 ratio and reflects the weight of tumor‐specific cfDNA in overall background cfDNA. (B) Segmentation‐based copy number analysis shows more prominent copy number changes in plasma than in serum. Most significant segment losses (arrows) were used to calculate tumor‐specific cfDNA difference between serum and plasma.

To determine whether plasma and serum showed any difference in tumor‐specific DNA content, we performed clustering analysis using GC‐corrected log2 ratio as input. Although the plasma and serum from the same patients clustered perfectly across all chromosome regions, there were clearly intensity differences in all regions showing copy number changes (Fig. 1A). Segmentation‐based copy number analysis further confirmed similar patterns of genomic gains/losses but different tumor cfDNA content (Fig. 1B). For example, patient 1 showed clear genomic loss at 5p and 8p in both plasma and serum samples. However, mean log2 ratio values at these segments were much smaller (hence, higher fraction of tumor‐derived cfDNA) in plasma than in serum. In all four cases, we observed consistent trend of higher tumor‐derived cfDNA content in plasma than in serum samples.

To accurately estimate tumor DNA content difference between serum and plasma, we selected mean value from a single most significant segment in each patient and calculated tumor‐specific DNA content. For patients 1 and 3, chromosome 8p showed most significant copy number loss. For patients 2 and 4, the most significant losses were 12q21 and 13q, respectively. Based on mean absolute log2 ratios at these selected genomic segments (Table 2), we estimated that tumor‐specific DNA in patient 1 accounted for 41.32% of plasma cfDNA and 21.08% of serum cfDNA, indicating that plasma contained 20.24% more tumor DNA fraction when compared to the matched serum sample. For other three paired samples, plasma also showed higher percentage of tumor DNA content (16.70%, 13.27%, and 8.51% more, respectively) than their corresponding serum samples. We also compared the PGA‐C scores between the four plasma–serum pairs. The PGA‐C score reflects the proportion of tumor‐specific DNA content in the overall background cfDNA. This analysis showed consistently higher PGA‐C scores in plasma than in matched serum samples (Table 2).

**Table 2:** Tumor‐derived cfDNA fraction and PGA‐C score in plasma and serum

	Patient 1		Patient 2		Patient 3		Patient 4	
	Plasma	Serum	Plasma	Serum	Plasma	Serum	Plasma	Serum
Selected segments	8p	8p	12q21	12q21	8p	8p	13q	13q
Log2 ratio (absolute)	0.77	0.34	0.45	0.16	0.72	0.43	0.43	0.27
Ratio (cfDNA/gDNA)	1.70	1.27	1.37	1.12	1.64	1.35	1.35	1.21
Tumor cfDNA (%)	41.32	21.08	27.02	10.32	39.17	25.90	25.79	17.28
PGA‐C score	273	130	291	201	213	140	122	55

### 3.5 Overall copy number changes

To evaluate overall genomic abnormalities, we performed log2 ratio‐based segmentation analysis using 1‐Mb genomic windows. This analysis showed subtle copy number changes in most samples tested, especially in patients with polyps and CRC stages I/II. From 79 patients with CRC, we detected significant copy number changes in 39 patients; most of them were from stages III and IV. The copy number changes were especially significant in two stage III patients and nine stage IV patients. The most common genomic changes in stage IV patients included whole chromosome gains on chr2, 7, 13, 20, partial chromosome gains at 8q11.2‐24.3, 12p11‐13.3, 13q12‐34, 20q11‐13.3, partial chromosome loss at 1p31.3‐36.23, 3p14.2, 4q13.2‐31.3, 8p12‐23, 17p13, 18q11.3‐22, and 22q11‐13.3. Detailed copy number changes are shown in Table S2.

**Figure 2:**
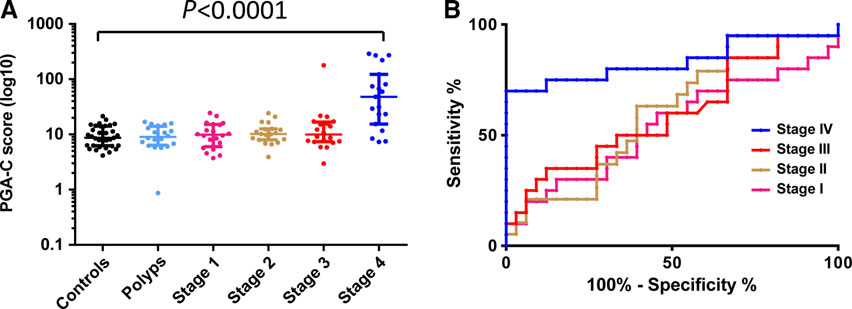
Association of PGA‐C score with disease status. (A) PGA‐C score increases with disease progression. (B) AUC analysis shows predictive performance of distinguishing CRC cases from healthy controls.

To estimate tumor‐specific cfDNA content among different disease stages, we applied the PGA‐C score algorithm. Our analysis showed that the average PGA‐C scores were 9.92 (healthy controls), 10.33 (polyp patients), 11.30 (CRC stage I), 11.83 (CRC stage II), 20.55 (CRC stage III), and 91.98 (CRC stage IV). Clearly, the patient groups (regardless of the disease stage) had higher average scores than the healthy controls. However, PGA‐C score was statistically higher in stage IV CRC patients only (Student's *t*‐test *P* = 3.51E‐05) (Fig. 2A). We also performed AUC analysis to evaluate diagnostic utility of PGA‐C score. By comparing to healthy controls, this analysis showed various discriminative ability with AUC of 0.53, 0.54, 0.61, 0.60, and 0.84 for patients with polyps, CRC stages I, II, III, and IV, respectively. Again, only stage IV was statistically significant (*P* < 0.0001) (Fig. 2B). This result indicated that PGA‐C score correctly classified stage IV cases as being at a higher risk than controls in 84% of case–control pairs.

### 3.6 Gene‐specific copy number changes

**Figure 3:**
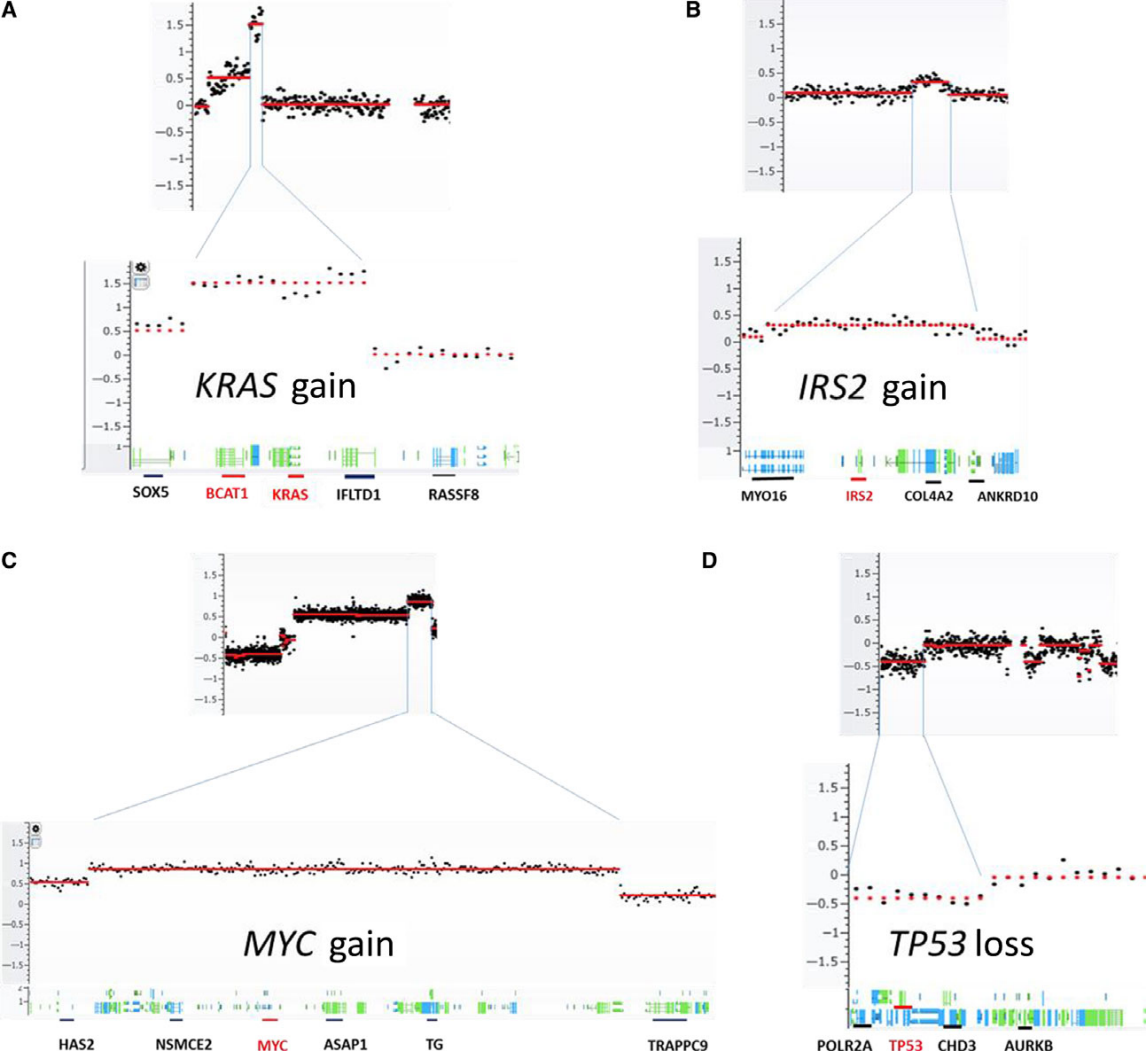
Representative gene regions with copy number changes. Each copy number change is illustrated in overall genomic view (upper panel) and detailed gene region view (lower panel).

**Figure 4:**
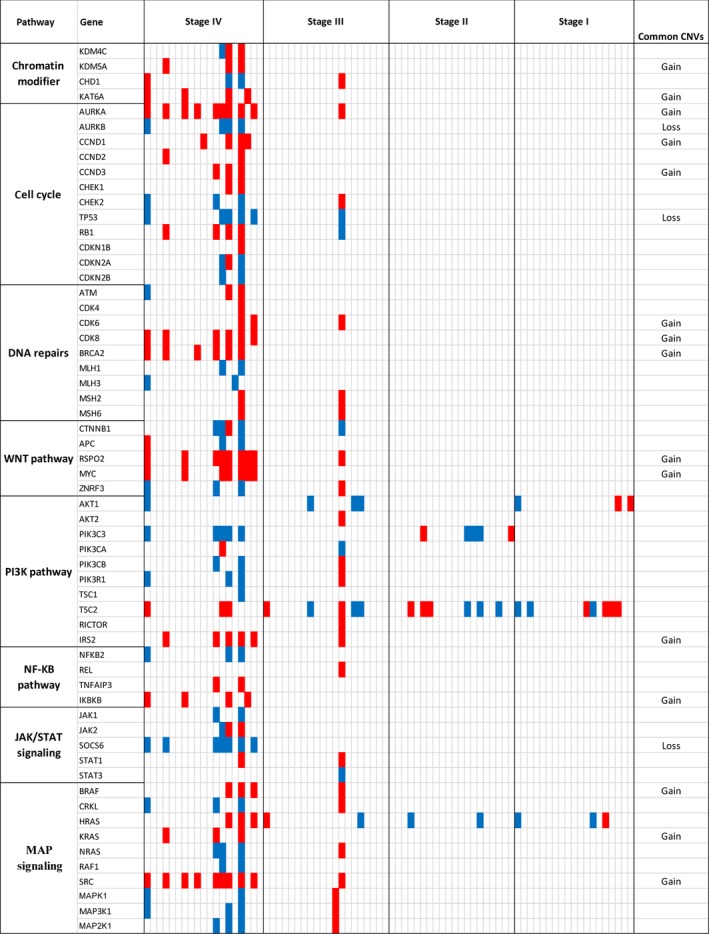
Oncoprint of copy number changes in critical genes of CRC‐related pathways. Red and blue represent gain and loss, respectively. Common CNVs are defined as ≥3 gains or losses (but not both) in all 79 cases tested.

To evaluate locus‐specific copy number changes, we narrowed the genomic segmentation size from 1‐Mb to 60‐Kb bin, which allowed more detailed analysis at gene loci. We focused on key genes in multiple signaling pathways implicated in CRC. This analysis showed that *BRAF*,* KRAS,* and *SRC* in MAP kinases pathway were commonly amplified in the cfDNA derived from stage III and IV CRC patients. In particular, we observed the copy number gain at *SRC* locus in 9 of 20 stage IV patients. We also observed frequent CNVs at *HRAS* locus from stage I to stage IV. However, the changes appeared to be a random event because both gains and losses were observed. For DNA damage/repair pathway, two genes *CDK8* and *BRCA2* showed significant genomic gains in six stage IV patients. In PI3K/AKT pathway, the most noticeable genomic gain was at locus of *IRS2*, where amplifications were found in five stage IV and one stage III samples. Although frequently detected at *TSC2* locus, the copy number changes were mixed with both deletions and amplifications. In cell cycle pathway, the most common gain was at *AURKA* locus, where nine amplifications in stage IV and one in stage III were observed. The most common loss was *TP53* gene, with deletions in five stage IV and one stage III patient. Other common changes included loss at *AURKB* and gains at *CCND1*. Additionally, *RSPO2*/*MYC* in the TGF‐β WNT signaling pathway and *KAT6A* in the chromatin modifier pathway showed copy number gains in eight and four stage IV patients, respectively. *SOCS6* in *JAK‐STAT* signaling pathway showed loss in seven stage IV patients. Representative copy number changes are presented in Fig. 3, and detailed changes for each individual genes and patients are shown in Fig. 4.

### 3.7 Locus‐specific copy number changes and overall survival

**Figure 5:**
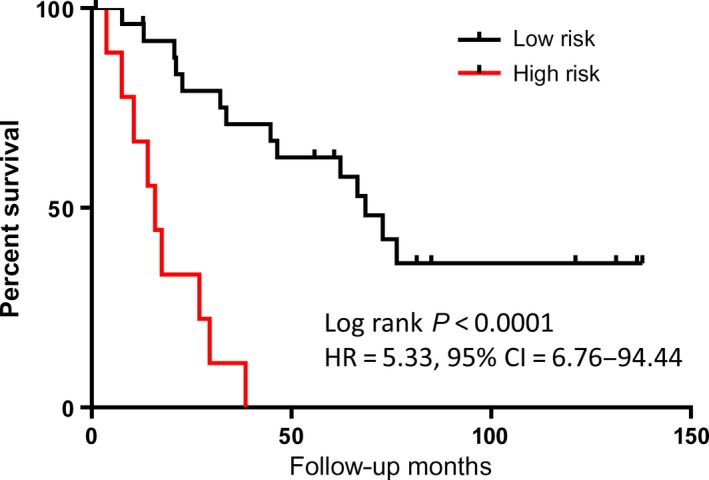
Kaplan–Meier analysis of overall survival. Six genomic segment‐based predictive model defines high‐risk group with median survival = 15.87 months and low‐risk group with median survival = 68.53 months in stage III‐IV CRC patients.

To associate the copy number changes with overall survival, we first performed segmentation analysis using 60‐kb genomic bin data and received a total of 3194 segments from chromosomes 1 to 22. We then extracted mean values from each segment and performed Cox regression analysis in stage III–IV patients. Of the 38 patients, 35 provided complete follow‐up data with vital status (23 deceased and 12 alive). This analysis revealed significant association of nine unique genomic segments with overall survival (FDR < 0.05). Among them were two unique regions on each of chromosomes 1, 3, and 22, and one unique region on each of chromosomes 2, 4, and 6. For example, the copy number loss at chr1: 2622000‐27180000 was associated with poor overall survival (HR = 0.82, 95% CI = 0.74–0.90, *P* = 5.91E‐5) (Table 3). To build a multivariate prediction model, we computed separate risk scores for different combination of the nine associated regions. We found that combination of six independent genomic regions gave the best discriminative performance of overall survival with high‐risk score group showing significantly shorter survival (HR = 5.33, 95% CI = 6.76–94.44, *P* < 0.0001). The median survival rate was at 68.53 months for the low‐risk group (*N* = 26) and 15.87 months for the high‐risk group (*N* = 9) (Fig. 5). The six independent genomic regions included chr1:26220001–26280000, chr2:159480001–159540000, chr3:9660001–9720000, chr4:49020001–49080000, chr6:97920001–97980000, and chr22:25740001–25800000.

**Table 3:** Genomic segments shsowing association with overall survival in stage III‐IV patients

chr	Start	Stop	Size	HR	95% CI	*P*‐value	FDR
1	26 220 001	27 180 000	960 000	0.82	0.74–0.90	5.91E‐05	2.89E‐02
1	120 780 001	121 560 000	780 000	0.81	0.72–0.90	1.58E‐04	2.89E‐02
2	159 480 001	160 200 000	720 000	1.34	1.14–1.58	3.32E‐04	4.24E‐02
3	9 660 001	10 020 000	360 000	0.69	0.57–0.83	8.85E‐05	2.89E‐02
3	14 340 001	14 700 000	360 000	0.93	0.90–0.97	2.61E‐04	3.62E‐02
4	49 020 001	49 140 000	120 000	0.78	0.69–0.89	1.62E‐04	2.89E‐02
6	97 920 001	99 060 000	1 140 000	1.29	1.14–1.46	3.21E‐05	2.89E‐02
22	17 100 001	18 660 000	1 560 000	0.91	0.87–0.96	5.05E‐04	4.98E‐02
22	25 740 001	25 980 000	240 000	0.92	0.88–0.96	1.72E‐04	2.89E‐02

## 4 Discussion

Emerging evidence has shown that cancer‐specific genetic variations are detectable in cfDNA derived from patients with cancer. The ability to detect genetic changes in cfDNA is often dependent on clinical stage, input cfDNA quantity, and detection technologies. In general, increased detection rates can be achieved with a higher input cfDNA and higher cancer stages (Bettegowda *et al*., 2014; Krishnamurthy *et al*., 2017). Newly developed technologies such as digital quantitative PCR and digital sequencing have improved the sensitivity of identifying rare genetic changes (Hudecova, 2015; Newman *et al*., 2016; Stahlberg *et al*., 2016). In this study, we applied low‐pass whole‐genome sequencing and evaluated copy number changes in the cohort containing healthy controls, patients with colorectal polyps, and patients with CRC. Our results demonstrate progressive CNV accumulation from stages I to IV and significant association of certain genomic loci with overall survival. This study further confirmed potential clinical applications of cfDNA‐based genetic variations as promising biomarker for cancer diagnosis and prognosis, especially for late‐stage cancers.

Although cancer‐derived cfDNA in peripheral blood has been extensively reported in recent years, the systematic evaluation of the difference between cfDNA of serum and plasma has not been performed. Because cfDNA concentration is generally very low in body fluids, obtaining the highest possible yield of cfDNA is important. However, the higher yield in serum may compromise the detectability of tumor‐derived cfDNA. Our data show that compared to plasma, serum contains much lower tumor‐specific DNA within cfDNA content as was the case in all serum–plasma pairs tested. Considering the need for high sensitivity, plasma is clearly a better choice than serum for the detection of tumor‐derived genetic changes.

As a heterogeneous disease, CRC displays significant molecular characteristics and clinicopathological features. Traditionally, sporadic CRC has been classified as hypermutated (16% of CRCs) and non‐hypermutated (84% of CRCs). The etiology for hypermutated tumors involves impairment of the DNA mismatch repair system, which may result either from germline mutations in MMR genes or from CpG island methylator phenotype via hypermethylation of one of the related DNA MMR genes. Non‐hypermutated tumors are more frequently associated with somatic CNVs at specific chromosome loci such as 8q gain and 8p loss, and common mutations in *APC*,* TP53*,* KRAS*,* SMAD4*, and *PIK3CA* (Carethers and Jung, 2015; Muller *et al*., 2016; Muzny *et al*., 2012). Through the sequencing‐based CNV analysis, we were able to detect the progressive accumulation of copy number changes in the critical genomic regions. Our cfDNA‐based study demonstrates clear copy number gains in 8q and losses in 8p in multiple patients, which is consistent with tumor tissue‐based CNV analysis in CRCs (Han *et al*., 2013; Hermsen *et al*., 2002).

Copy number changes at critical regions have shown potential clinical utilities to predict treatment response and clinical outcomes (Wang *et al*., 2016). Several studies have shown that 20q gains and 1p losses are associated with poor prognosis in patients with CRC (Knosel *et al*., 2004; Ogunbiyi *et al*., 1997; Postma *et al*., 2007). Copy number gain of *MYC* gene at 8q24 has been frequently reported in CRC (Eldai *et al*., 2013). A recent study examined 367 patients with CRC using dual‐color silver in situ hybridization and observed copy number gain at the *MYC* locus as an independent factor for poor prognosis (Lee *et al*., 2015). *KRAS* gene at 12p12 has long been studied as a crucial oncogene, and tumor cells with wild‐type *KRAS* may benefit from anti‐EGFR therapy. Amplification of *KRAS* gene has also been identified as a crucial factor that leads to RAS/mitogen‐activated protein kinase activation. *KRAS* gene copy number loss in tumor DNA is associated with better treatment response to anti‐EGFR drugs even in the presence of *KRAS* mutation in the tumor. On the other hand, copy number gain of *KRAS* predicts resistance to drugs independent of mutational status (Mekenkamp *et al*., 2012). Therefore, it is worthwhile to detect both copy number changes and gene mutations at these critical gene loci in the plasma to guide selection of therapeutic treatments.

Although we successfully identified significant copy number changes in cfDNA, the study has shown some limitations. First, we were not able to evaluate either the mutation status in these major genes or methylator phenotypes, which are two important genetic and epigenetic events involved in CRC initiation and progression. Second, the copy number analysis is based on low‐pass whole‐genome sequencing. Although able to detect gross copy number changes, it is not sensitive to detect low copy number gain/loss and smaller genomic changes. Increase in sequencing depth may be required to detect rare genomic events. Third, due to low tumor DNA component in early‐stage cancer, the copy number analysis using plasma cfDNA is currently not ready for CRC screening at the potentially curable stage. Lastly, due to our smaller sample size, our findings require validation in larger cohorts. Nevertheless, our study provides strong evidence that plasma‐based cfDNA tests have a great potential to be used as a measurable classifier for disease diagnosis and clinical outcome assessment in advanced CRC patients.

## Author contributions

JL, RLD, and SX performed experimental tests and initiated the manuscript. HZ, MD, and CCH performed data analysis. BRD and LB provided clinical data. LW initiated and directed the study.

## Data accessibility

## Supporting information


**Fig. S1.** cfDNA concentrations in serum, plasma and among different stages.
**Fig. S2.** Quality control of plasma cfDNA sequencing libraries.
**Fig. S3.** Clustering analysis of six technical replicates.

**Table S1.** Sequencing raw read counts and mappable read counts.

**Table S2.** Overall plasma copy number changes.
