# Supplementary figures and images for: Cell‐free DNA copy number variations in plasma from colorectal cancer patients
Source: Mol Oncol. 2017 Jun 6;11(8):1099–111. doi: 10.1002/1878-0261.12077 (PMC5537711; doi:10.1002/1878-0261.12077)

**Supplementary Figure S1.**


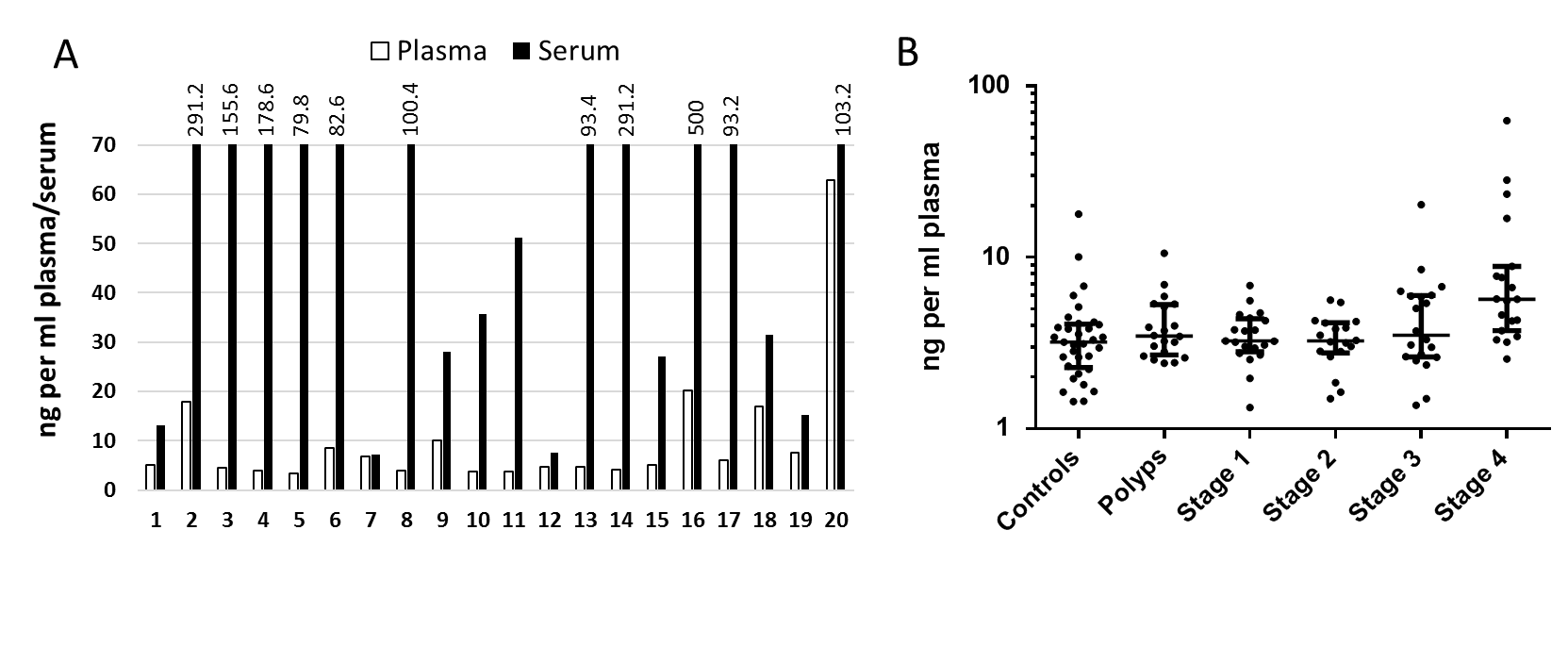


**Supplementary Figure S2.**


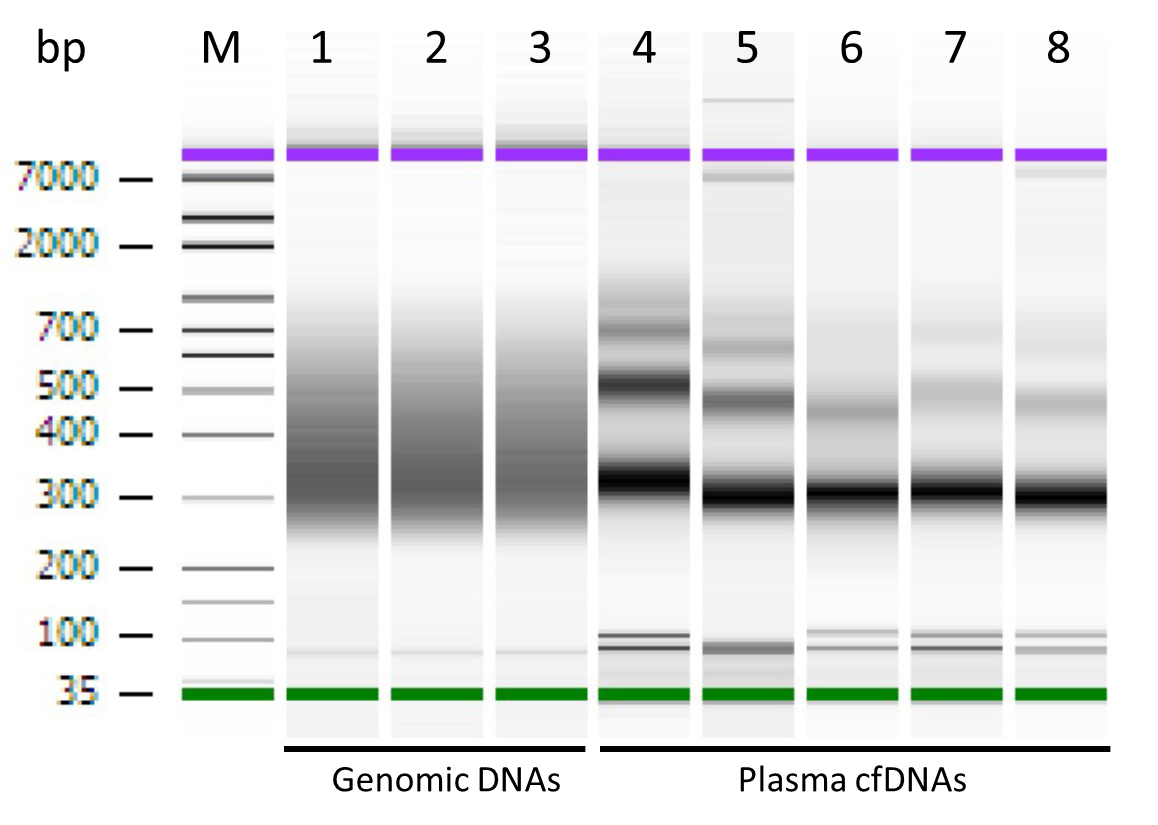


**Supplementary Figure S3.**


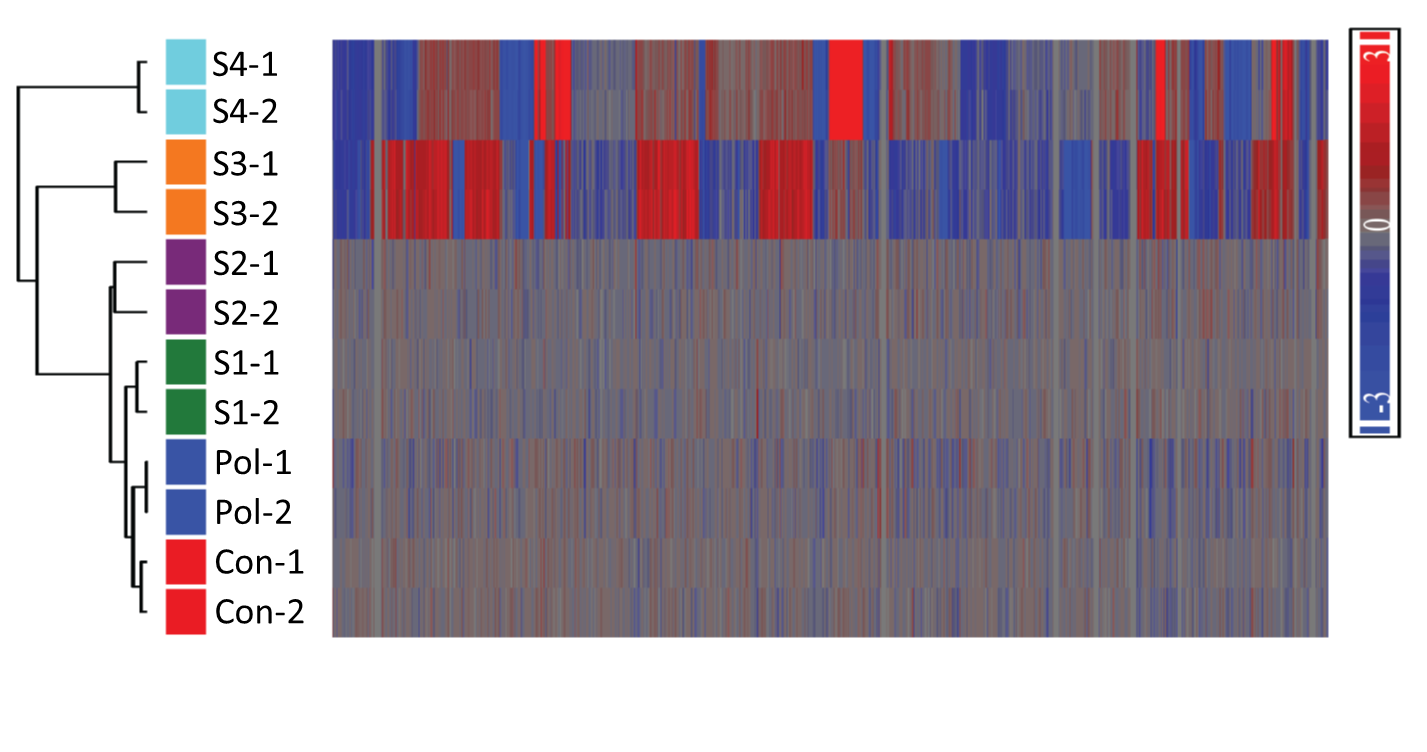

Supplement: Supplementary file 1 — Fig. S1. cfDNA concentrations in serum, plasma and among different stages. Fig. S2. Quality control of plasma cfDNA sequencing libraries. Fig. S3. Clustering analysis of six technical replicates. [file MOL2-11-1099-s001.docx]
